# Effect of Steroids on Anti-nephrin Autoantibodies and B cells in Minimal Change Disease

**DOI:** 10.1016/j.ekir.2026.103794

**Published:** 2026-01-23

**Authors:** Felicitas E. Hengel, Asma Beldi-Ferchiou, Hamza Sakhi, Nastassia Liaukouskaya, Silke Dehde, Alexandre Karras, Emmanuel Esteve, Julie Oniszczuk, Sabine Le Gouvello, Luis Herrera-Marcos, Nizar Joher, Eric Daugas, Mohamad Zaidan, Khalil El Karoui, Bertrand Knebelmann, Myriam Dao, Anissa Moktefi, Dil Sahali, Tobias B. Huber, Mario Ollero, Nicola M. Tomas, Vincent Audard

**Affiliations:** 1 III Department of Medicine, University Medical Center Hamburg-Eppendorf, Hamburg, Germany; 2 Hamburg Center for Kidney Health, University Medical Center Hamburg-Eppendorf, Hamburg, Germany; 3 Hamburg Center for Translational Immunology, University Medical Center Hamburg-Eppendorf, Hamburg, Germany; 4 Institut National de la Santé et de la Recherche Médicale (INSERM) U955, Institut Mondor de Recherche Biomédicale, Universite Paris Est Créteil, Créteil, France; 5 Department of Biological Immunology and Hematology, Assistance Publique-Hôpitaux de Paris, Henri Mondor University Hospital, Créteil, France; 6 Nephrology and Renal Transplantation Department, Assistance Publique-Hôpitaux de Paris, National Rare Disease Center Idiopathic Nephrotic Syndrome, Henri Mondor University Hospital, Créteil, France; 7 Nephrology Department, Assistance Publique-Hôpitaux de Paris, Hôpital Européen Georges Pompidou, Paris Cité University, Paris, France; 8 Nephrology Department, Assistance Publique-Hôpitaux de Paris, Tenon Hospital, Sorbonne University, INSERM U1115 CORAKID, Paris, France; 9 Nephrology Department, Assistance Publique-Hôpitaux de Paris, Bichat Hospital, Paris Cité University, INSERM U1149, Paris, France; 10 Nephrology Department, Assistance Publique-Hôpitaux de Paris, Kremlin Bicêtre Hospital, Saclay University, Paris, France; 11 Nephrology Department, Assistance Publique-Hôpitaux de Paris, Necker Hospital, Paris Cité University, Paris, France; 12 Pathology Department, Assistance Publique-Hôpitaux de Paris, Henri Mondor University Hospital, Créteil, France

**Keywords:** anti-nephrin antibodies, minimal change disease, nephrotic syndrome, plasmablasts, steroids

## Abstract

**Introduction:**

The identification of autoantibodies against nephrin, a key signaling protein of the podocyte slit diaphragm, and their pathogenic role in patients with minimal change disease (MCD) has substantially changed our understanding of primary podocytopathies. However, details on underlying immune pathophysiological mechanisms such as the relation of anti-nephrin autoantibodies with immune cell subsets and therapy response remain to be determined.

**Methods:**

In this prospective study, we evaluated blood samples from adults with newly diagnosed, biopsy-proven MCD for circulating anti-nephrin antibodies using immunoprecipitation (IP) and B-cell subpopulations by fluorescence-activated cell sorting. Samples were collected at diagnosis (t_0_) and after 8 weeks of steroid therapy (t_1_).

**Results:**

We detected anti-nephrin antibodies in 12 of 17 (70.6%) therapy-naïve patients with MCD at t_0_. All 12 patients (100%) positive for anti-nephrin antibodies achieved complete remission with no anti-nephrin antibodies detectable after 8 weeks of steroid treatment (t_1_). Two of 5 anti-nephrin negative patients (40%) did not reach clinical remission at t_1_. Anti-nephrin positive patients exhibited a larger fraction of plasmablasts than anti-nephrin negative patients at t_0_, albeit not statistically significant. Plasmablasts, naïve B cells, and transitional B cells significantly decreased, whereas marginal zone–like B cells increased within 8 weeks of steroid treatment in anti-nephrin positive patients (t_1_).

**Conclusion:**

Our study shows, for the first time, a therapeutically relevant short-term effect of steroids on the B-cell compartment and anti-nephrin antibody levels, explaining the high clinical response rate in patients with anti-nephrin-associated MCD.

## Graphical abstract

**Figure:**
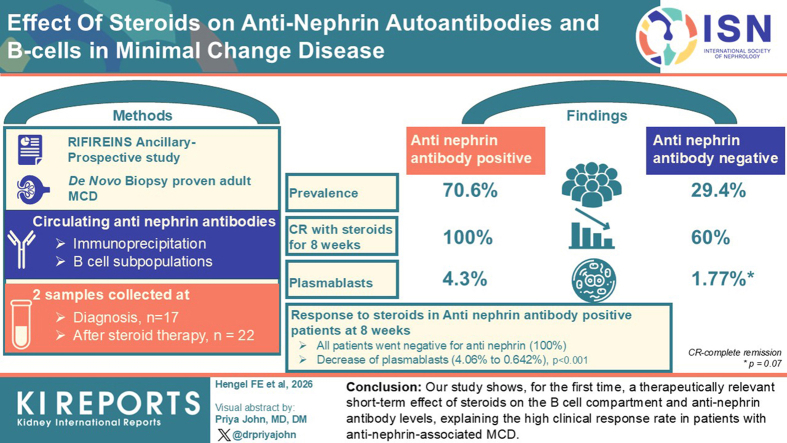

MCD is a glomerular disease characterized by the clinical phenotype of nephrotic syndrome and defined by the histological pattern of unaltered glomerular morphology on light microscopy and extensive foot process effacement on electron microscopy.^1^^,^^2^ Until recently, it was postulated that MCD is initiated by an immune dysregulation, leading to the release of permeability factor(s), possibly T or B lymphocyte–derived, which disrupt(s) the glomerular filtration barrier by inducing podocyte injury with subsequent proteinuria.^3^

The recent discovery of antibodies against nephrin, a key structural protein of podocytes, as a pathogenic factor and biomarker of a subset of patients with MCD provides new insights into the pathogenesis for a large part of affected patients.^4^, ^5^, ^6^ Anti-nephrin antibodies are found in 44% of adult patients with a histological diagnosis of MCD, show a strong correlation with disease activity (i.e., presence of antibodies during initial diagnosis and relapse, and absence in remission) and their prevalence increases up to 69% if only immunosuppression-naïve patients with active nephrotic syndrome are analyzed.^5^

Currently, steroid therapy represents the cornerstone of initial treatment for the majority of both adult and pediatric patients with MCD.^7^ Interestingly, pediatric patients who are positive for anti-nephrin antibodies tend to respond well to steroid treatment, as indirectly evidenced by an antibody prevalence of 82% in steroid-sensitive as opposed to 14% in steroid-resistant cases of children with active nephrotic syndrome.^8^ However, how steroids act on anti-nephrin autoantibodies and immune cells to support clinical remission in the context of anti-nephrin-mediated *de novo* MCD remains to be determined.

Here, we longitudinally characterized anti-nephrin antibodies and B-cell subsets in a prospective cohort of adult patients with a first episode of biopsy-proven MCD at the time of renal biopsy and at 8 weeks after steroid initiation.

## Methods

### Patients and Biological Collection

**Figure 1:**
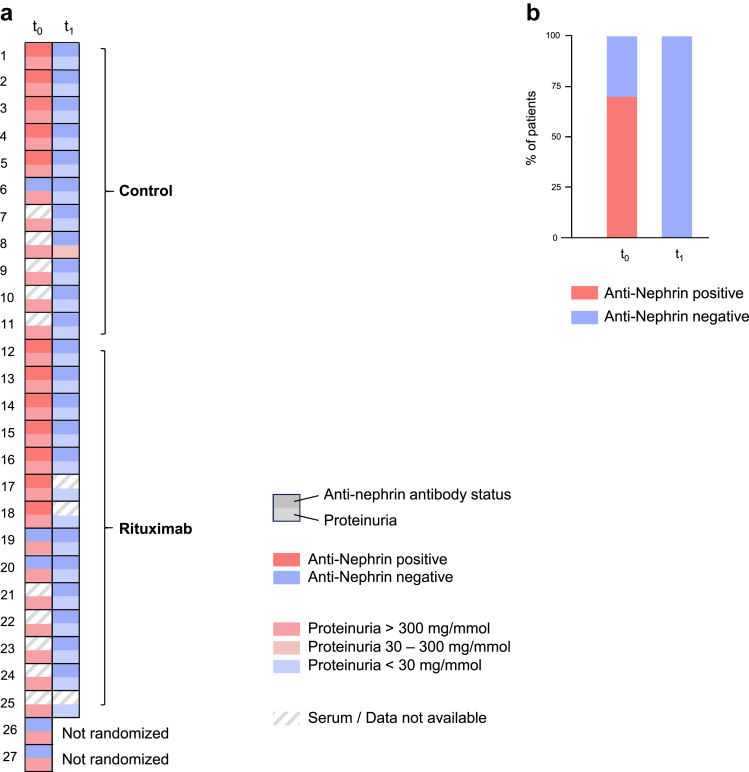
Anti-nephrin autoantibody status at diagnosis and complete remission after steroid therapy. (a) Heat map of anti-nephrin antibody status at minimal change disease diagnosis (t_0_) and at randomization after 8 weeks of steroid therapy (t_1_), Each row represents 1 patient, and each column corresponds to 1 time point. All patients who reached remission after 8 weeks of steroid therapy were randomized into 2 groups (control [*n* = 11] and rituximab [*n* = 14]) independently of serum availability or anti-nephrin status. (b) Positive rates of anti-nephrin antibodies in patients at t_0_ (*n* = 17) and t_1_ (*n* = 22).

This was an ancillary study (biological collection) from the ongoing prospective study entitled “Rituximab From the First Episode of Minimal Change Disease for Preventing Relapse Risk in Adult Patients: a Multicenter Randomized Controlled Trial” (RIFIREINS study ClinicalTrials.gov: NCT03970577). Baseline samples were available from 17 patients for anti-nephrin antibody analysis at t_0_ (i.e., at study inclusion), before initiation of steroid therapy, and 22 samples were obtained at t_1_ (i.e., after 8 weeks of high-dose steroid treatment), corresponding to a total of 39 blood samples from 26 adult (aged ≥ 18 years) patients with *de novo* diagnosis of histologically-proven MCD enrolled from Île-de-France centers (27 were initially included, but 1 sample was not analyzable due to a technical issue) (Figure 1, and Supplementary Figure S1). Alongside, we conducted a comprehensive analysis of B-lymphocyte subpopulations. Patients were referred to 1 of 6 French university hospital nephrology departments in the Ile de France area (Henri Mondor, Tenon, Necker Enfants Malades, Hôpital Européen Georges Pompidou, Kremlin Bicètre, and Bichat) between January 2021 and May 2023 for exploring a first episode of nephrotic syndrome (urine protein-to-creatinine ratio ≥ 300 mg/mmol and albumin level < 30 g/l), requiring a renal biopsy. Renal biopsy specimen demonstrated the typical pathological features of MCD by light microscopy and immunofluorescence study. All patients were included in the ancillary study (biological collection for blood and urine samples biobanking) backed up by the ongoing randomized RIFIREINS trial. The main objective of RIFIREINS trial is to test the efficacy of rituximab with definitive steroid withdrawal after a total of 9 weeks of treatment compared with the standard regimen of oral steroids alone (progressively tapered within 24 weeks) to prevent relapse after 12 months of follow-up in adult patients with *de novo* MCD. After 8 weeks of steroid therapy, patients who reached complete remission of nephrotic syndrome are randomized into 2 groups as follows: (i) rituximab: 2 injections separated by 1 week 375 mg/m^2^ with definitive steroid withdrawal in 1 week (experimental group); and (ii) exclusive oral steroid (prednisone) therapy (progressively tapered with the same procedure for all patients) for a total exposure of 24 weeks (including initial oral steroid therapy administered during 8 weeks) (control group).

The ancillary study aims to investigate the relationship between lymphocyte subpopulations and treatment response at the time of inclusion (before initiation of steroid therapy, t_0_) and following 8 weeks of steroids (at the time of randomization if the patient exhibited complete remission of nephrotic syndrome defined as albumin level > 30 g/l and urine protein-to-creatinine ratio < 30 mg/mmol, t_1_). All patients included in the current study gave their signed consent to participate in the RIFIREINS trial including the ancillary study. This study was performed in accordance with the ethical standards of the Helsinki Declaration and with the approval of the *Comité de Protection des Personnes Sud Ouest et Outre mer* (N° EUDRACT 2018-003437-15, EU CT number: 2024-516102-36-00).

### Detection of Anti-nephrin Antibodies

#### IP and Western blotting

Anti-nephrin antibodies were determined by IP using recombinant human nephrin and subsequent Western blotting as described previously.^5^ Briefly, patient serum was incubated with recombinant nephrin overnight at constant rotation at 4 °C, and MabCaptureC High Capacity Protein A Resin (Thermo Fisher Scientific, Bleiswijk, Netherlands) was added for 2 hours to bind patient IgG. The resin was washed, and bound proteins were eluted under denaturing conditions. Eluates were electrophoresed using SDS-PAGE, transferred to methanol-soaked PVDF membranes (EMD Millipore), which were blocked with 3% dry milk, and precipitated nephrin was detected with a commercial anti-nephrin antibody (polyclonal sheep anti-nephrin antibody, R&D Systems; AF4269, Abingdon, United Kingdom).

#### Quantitative IP and Enzyme-Linked Immunosorbent Assay

A 2-step procedure of IP followed by enzyme-linked immunosorbent assay (ELISA)-based quantification of immunoprecipitated recombinant nephrin was applied for quantification of anti-nephrin autoantibody titers.^5^ Sixty μl patient serum was mixed with 150 ng of recombinant Twin-Strep-tagged nephrin in RIPA buffer, incubated at constant rotation over night at 4 °C and exposed to MabCaptureC High Capacity Protein A Resin (Thermo Fisher Scientific) for 2 hours. The resin was washed using Pierce Spin Columns (Thermo Fisher Scientific) twice in RIPA buffer followed by 4 washes in phosphate buffered saline with 0.2% Tween 20 and once in phosphate buffered saline. Acid protein elution was done using IgG elution buffer (Thermo Fisher Scientific) at pH of 2.8 and neutralization with 1 M Tris pH of 9. Next, immunoprecipitated Twin-Strep-tagged nephrin was quantified using a Streptactin-coated microplate (IBA Lifesciences GmbH). Wells were incubated with the eluate diluted in postcoat buffer (triethanolamine-buffered saline with bovine serum albumin, Sigma-Aldrich) with 0.05% Tween 20 for 2 hours at 20 °C. Wells were washed 4 times with triethanolamine-buffered saline–T (Sigma-Aldrich) and incubated with anti-nephrin antibody (polyclonal sheep anti-nephrin antibody, R&D Systems; AF4269, diluted 1:1000 in postcoat buffer with 0.05% Tween 20) overnight on a rocking platform at 4 °C. Wells were washed 4 times and incubated with 100 μl of HRP-conjugated anti-shIgG (Jackson ImmunoResearch, diluted 1:10,000 in postcoat buffer with 0.05% Tween 20) for 2 hours at 20 °C. Wells were washed again and 100 μl TMB ELISA peroxidase substrate solution (Avia Systems Biology) was applied for 5 minutes at 20 °C, followed by 100 μl of 1 mol/l H_3_PO_4_ solution to stop the substrate reaction. Absorbance at 450 nm was determined using an ELISA reader (PlateDirect A96, Mettler Toledo). Quantification was done in duplicates, background subtraction was performed using incubation with 100 μl polyclonal sheep IgG (Sigma-Aldrich, 0.2 μg/ml in postcoat buffer with 0.05% Tween 20) in place of polyclonal sheep anti-nephrin antibody, and relative units (RU/ml) were determined in regard to a standard curve of serial dilutions of recombinant human nephrin. The second last serial dilution of recombinant human nephrin corresponding to a relative unit of 25 RU/ml served as a cut-off for a positive anti-nephrin signal.

### Flow Cytometry Analysis of B Cells

Blood samples were prospectively collected in ethylenediaminetetraacetic acid tubes and shipped at room temperature. Immunostaining for B-cell subsets was performed within 2 hours on whole blood samples with the standardized DuraClone IM B Cells technique (Beckman-Coulter) according to the manufacturer’s recommendation. Each DuraClone IM B Cell tube contains a cocktail of 8 conjugated monoclonal antibodies: IgD-FITC, CD21-PE, CD19-ECD, CD27-PC7, CD24-APC, CD38-APC-A750, IgM-Pacific Blue, and CD45-Krome Orange. Whole blood (300 μl) was washed twice in 1X phosphate-buffered saline, supernatant was then discarded. The pellet was resuspended in 300 μl of 1X phosphate buffered saline and 100 μl of the washed whole blood were added to a reagent tube and incubated in the dark for 15 minutes at room temperature. After red blood cell lysis (VersaLyse Solution, Beckman-Coulter), washed samples were immediately acquired on a Navios 10-colour flow (Beckman-Coulter) and data were analyzed with Kaluza version 2.1 software (Beckman-Coulter). B cell gating strategy is shown in Supplementary Figure S2.

### Statistical Analysis

Categorical and continuous variables were expressed as count (percentage) and median (interquartile range) or mean (± SD), respectively. When appropriate, chi-square or Fisher exact tests were used for categorical comparison, and Mann–Whitney or Wilcoxon signed rank test (for paired tests) for continuous variables. Correlation analyses were conducted using Spearman’s method. A *P*-value < 0.05 was considered significant. Tests were 2-tailed and not adjusted for multiplicity. Statistical analyses were carried out using R 3.6.2 and GraphPad Prism 9.0 (GraphPad Software Inc.).

## Results

### Detection of Anti-nephrin Antibodies

Twenty-seven patients with newly diagnosed biopsy-proven MCD were included (17 men [63%]) with median age of 40 [30–53.5] years). Six (22%) had a history of hypertension and 2 (7%) had diabetes mellitus. At baseline (t_1_), median urine protein-to-creatinine ratio was 649 (432–839) mg/mmol, median serum albumin level was 15 g/l (11–20) and median creatinine level was 85 (66–97) μmol/l. After 8 weeks of steroid therapy (1 mg/kg/d), 25 of 27 patients achieved clinical remission. At t_0_ and t_1_, serum samples were available from 17 and 22 patients, respectively (Figure 1a). At t_0_, anti-nephrin antibodies were detected in 12 of 17 patients (70.6%) (Figure 1a and b, Table 1). We compared the clinical and biological characteristics of anti-nephrin antibodies positive (*n* = 12) and negative (*n* = 5) patients at study inclusion (Table 1). We found a trend toward more severe hypoalbuminemia in patients with positive anti-nephrin antibodies than in patients with negative anti-nephrin antibodies (13.5 (10–17) g/l and 20 (20–23) g/l, respectively, *P =* 0.06). Urine protein-to-creatinine ratio levels were not statistically different (788.6 (466.64–878.8) mg/mmol vs. 620 (512.5–697.69) mg/mmol, respectively; *P =* 0.3) (Table 1). Next, we used a quantitative IP ELISA to assess anti-nephrin levels in the 17 patients at t_0_. Nine patients were positive with titers above the predefined cutoff of 25 RU/ml, whereas the 3 patients who were positive by IP-Western Blotting–positive had titers of 5, 10, and 24 RU/ml. Considering the 12 IP-Western Blotting–positive patients, the median IP-ELISA titer at diagnosis was 93 (27.96–127.75) RU/ml; whereas for the 5 IP-Western Blotting–negative patients the median was 5 (0–5.54) RU/ml, *P* = 0.006 (Table 1, Supplementary Figure S3A and B). All 12 anti-nephrin positive patients went into complete remission after 8 weeks of steroids with no detectable anti-nephrin antibodies. Accordingly, the median anti-nephrin titer decreased from 93 (27.96–127.75) RU/ml at diagnosis to 0 (0–0) RU/ml at week 8 (Supplementary Figure S3A). In contrast, only 3 out of 5 patients (60%) with anti-nephrin negative assessment at inclusion achieved complete remission after 8 weeks of steroid treatment (*P =* 0.07). Because of absence of complete remission, the 2 remaining anti-nephrin negative patients were not randomized to receive either 2 injections of rituximab separated 1 week apart or a steroid regimen tapered progressively (Figure 1a and b, Table 1). Whole-exome sequencing was performed in the 2 steroid-resistant patients to rule out genetic causes of nephrotic syndrome and was negative.

**Table 1:** Baseline characteristics of minimal change disease patients depending on anti-nephrin antibody detection at the time of diagnosis (before steroid initiation)

	Total (*N* = 17)	Anti-nephrin (−) (*n* = 5)	Anti-nephrin (+) (*n* = 12)	*P*-value
Age (yr), median (IQR)	35.0 (25.0–47.0)	43.0 (25.0–52.0)	34.5 (26.0–43.3)	0.8
Sex, male, *n* (%)	9 (53)	2 (40)	7 (58)	0.6
UPCR (mg/mmol), median (IQR)	705.4 (461.6–864.1)	620.0 (512.5–697.69)	788.6 (466.64–878.8)	0.3
Albumin level (g/l), median (IQR)	17.0 (11.0–20.0)	20.0 (20.0–23.0)	13.5 (10.0–17.0)	0.06
Creatinine level (μmol/l), median (IQR)	86.0 (69.0–99.0)	85.0 (69.0–91.0)	88.0 (71.3–103)	0.6
eGFR at diagnosis (ml/min per 1.73 m^2^), median (IQR)	89.0 (79.0–106)	101 (89.0–106)	85.5 (77.8–115)	0.8
Anti-nephrin Abs level (RU/ml), median (IQR)	29 (5.5–112.58)	5 (0–5.54)	93 (27.96–127.75)	0.006
Complete remission after 8 wks of steroid therapy, *n* (%)	15 (88.2%)	3 (60%)	12 (100%)	0.07

Abs, antibodies; eGFR, estimated glomerular filtration rate; IQR, interquartile range; UPCR, urine protein creatinine ratio.

### Distribution of B-Lymphocyte Subset and Correlation With Anti-nephrin Antibodies Positivity

**Figure 2:**
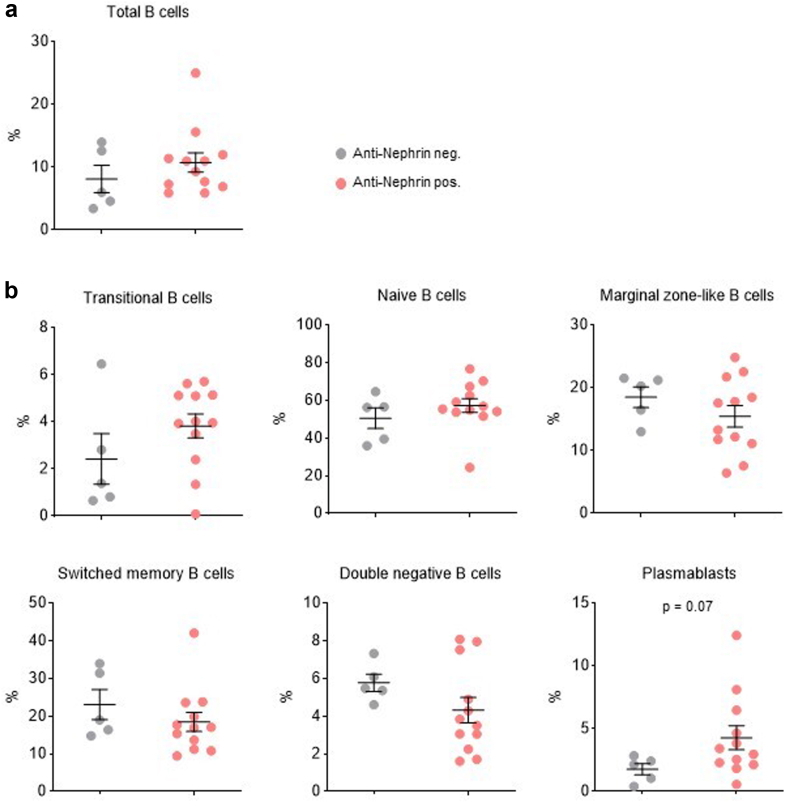
Analysis of B-cell subsets at the time of inclusion (t_0_, before steroid therapy initiation) according to anti-nephrin autoantibody status (positive anti-nephrin antibodies, *n* = 12; negative anti-nephrin antibodies, *n* = 5). Results are given as % among (a) lymphocytes or (b) % among B cells. Data are shown as scatterplots with black bars indicating mean ± SEM.

**Figure 3:**
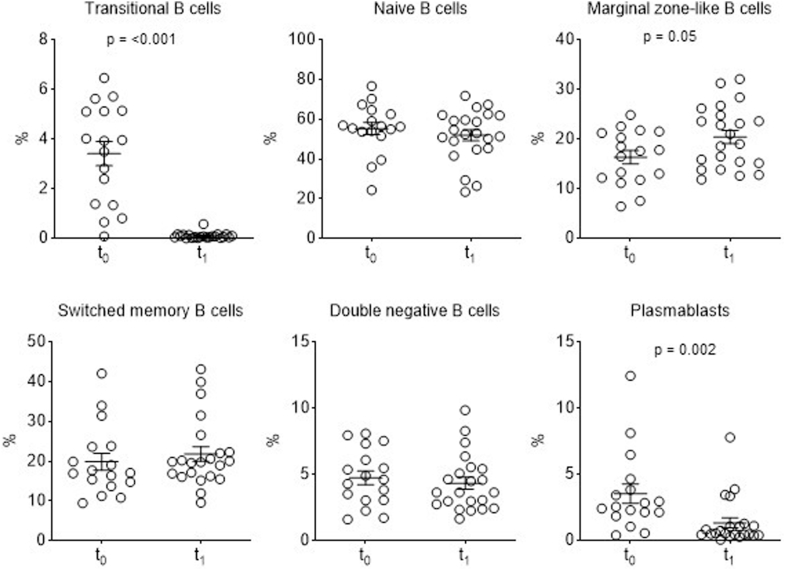
Analysis of B-cell subsets evolution before (t_0_) and after (t_1_) 8 weeks of steroid therapy (across all patients tested for anti-nephrin autoantibodies (*n* = 39). Results are given as % among B cells. Data are shown as scatterplots with black bars indicating mean ± SEM.

**Figure 4:**
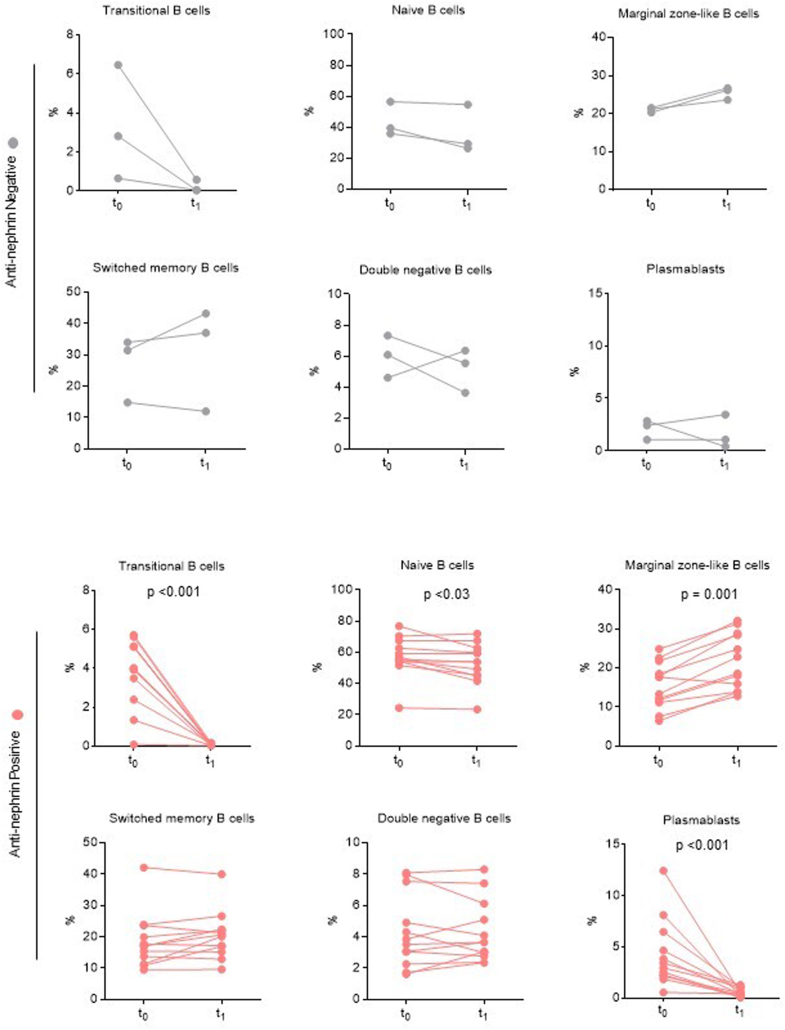
Analysis of B-cell subsets evolution before (t_0_) and after (t_1_) 8 weeks of steroid therapy according to anti-nephrin autoantibody status. (positive anti-nephrin antibodies, *n* = 10; negative anti-nephrin antibodies *n* = 3). Results are given as % among B cells.

We first investigated whether B-cell subsets differ between patients with positive and negative anti-nephrin antibodies at t_0_ before steroid initiation. Total B cells did not differ between both groups (Figure 2a). We found a trend toward higher mean percentage of plasmablasts in patients with positive anti-nephrin antibodies (*n* = 12) (4.3% ± 3.3) compared with patients negative for anti-nephrin antibodies (*n* = 5) (1.77% ± 1) (*P =* 0.07) (Figure 2b). We next studied the correlation between plasmablast levels and anti-nephrin titers at diagnosis. We observed a trend toward but not statistically significant positive correlation between plasmablast levels and anti-nephrin titers (*r* = 0.45; *P* = 0.06) (Supplementary Figure S3C). No significant differences between anti-nephrin positive and anti-nephrin negative patients were observed for other B-lymphocyte subsets (Figure 2b). Second, we compared B-cell subpopulations between the 17 patients with available samples at inclusion (t_0_) and the 22 patients in remission of nephrotic syndrome after 8 weeks of steroids treatment (t_1_) (Figure 3). Of note, samples at t_1_ were not available for 2 patients (P26 and P27) because of study exclusion upon persistent nephrotic syndrome (Figure 1a). We found that patients displayed a marked reduction of mean percentage of plasmablasts and transitional B cells after 8 weeks of steroid therapy and upon nephrotic syndrome remission (plasmablasts: 3.54% (± 3) and transitional B cells 3.4% (± 2) before steroid initiation and 1.34% (± 1.8) and 0.1% (± 0.12) after 8 weeks of steroids, *P* = 0.002 and *P <* 0.001, respectively) (Figure 3). By contrast, the mean percentage of marginal zone–like B cells was higher after 8 weeks of steroid treatment (t_1_) (20.40% ± 6.23) compared with t_0_ (16.34% ± 5.46) but did not reach statistical significance (*P =* 0.05). The evolution of B-lymphocytes subpopulations between t_0_ and t_1_ depending on anti-nephrin Abs status is shown in Figure 4. Among anti-nephrin positive patients, remission of nephrotic syndrome and disappearance of anti-nephrin antibodies between t_0_ and t_1_ were associated with a significant decrease in the mean percentage of plasmablasts from 4.06% (± 3.36) to 0.642 (± 0.40) (*P* < 0.001), of transitional B cells (from 3.68% [± 3.36] to 0.08% [± 0.05]; *P <* 0.001) and of naïve B cells from 57.8 (± 14.2) to 52.5% (± 14.45) (*P <* 0.03). By contrast, the mean percentage of marginal zone–like B cells significantly increased significantly from t_0_ to t_1_ (15.58 (± 6.41) to 21.41(± 7.46), *P =* 0.001). In patients positive for anti-nephrin antibodies, the percentage of switched memory B cells and double negative B cells remained unchanged between t_0_ and t_1_ (Figure 4). Among the 3 patients who tested negative for anti-nephrin antibodies and who achieved complete remission, no significant differences before and after steroid treatment were observed in B cell population, particularly within the plasmablast compartment (Figure 4).

## Discussion

The successful use of B-cell–depleting agents to maintain long-term remission^9^ along with the recent identification of antibodies targeting the podocyte protein, nephrin,^4^^,^^5^ support a crucial role of humoral immunity in the pathogenesis of primary podocytopathies.^1^^,^^3^^,^^10^ In the ancillary study of the ongoing RIFIREINS trial, we assessed anti-nephrin antibody prevalence and the effects of initial steroid therapy administered during 8 weeks, on antibody levels and B-cell subsets in adult patients with newly diagnosed MCD.

We found anti-nephrin antibodies in 70.6% of adult patients with a first episode of MCD prior to immunosuppression, a percentage comparable with the previous cohort study by Hengel *et al.*^5^ (69%) and slightly higher than the data of Shu *et al.*^11^ (53%). In our study, anti-nephrin positive patients had lower serum albumin than anti-nephrin negative patients at t_0_, albeit not statistically significant. Further clinical and biological characteristics did not differ between both groups, possibly because of limited statistical power. These data are consistent with previous results indicating that the presence of these antibodies is associated with more severe nephrotic syndrome.^5^ The effect of steroid treatment as first line therapeutic option on anti-nephrin antibodies among patients with MCD remains to be assessed. The RIFIREINS trial design, in which all adult patients are first treated with a high dose of oral steroids before randomization to receive either rituximab or a progressive reduction in the dose of steroids, enabled us to prospectively evaluate the evolution of anti-nephrin antibodies after 8 weeks of steroid treatment. At this time point, complete remission of nephrotic syndrome was associated with the disappearance of anti-nephrin antibodies in all initially anti-nephrin positive patients, whereas only 60% of initially anti-nephrin negative patients (3/5) achieved complete remission after 8 weeks of steroids. This result is in line with recent findings indicating that the prevalence of anti-nephrin antibodies is higher in immunosuppression-responsive forms of idiopathic nephrotic syndrome (INS) in children than those with therapy-resistant forms^8^ and further strengthens the role of anti-nephrin antibodies as a valuable biomarker in primary podocytopathies.^12^ Although the presence of anti-nephrin autoantibodies could in general indicate a favorable treatment response, recent studies suggest that anti-nephrin antibodies may be associated with a higher risk of relapsing disease after transplantation.^13^^,^^14^ Anti-nephrin antibodies differ from other autoantibodies encountered in distinct antibody-mediated podocytopathies such as membranous nephropathy by their presumably low circulating levels, which could contribute to their rapid disappearance after steroid therapy. Second, their mechanism of action through direct interference with the nephrin function and signaling, leading to a more direct correlation between autoantibody presence and podocyte dysfunction, and fitting with the phenotype of abrupt onset and rapid remission of nephrotic syndrome usually observed in this disease.

One of the strengths of our study is the characterization of B-lymphocyte subsets in parallel with anti-nephrin antibodies before and after specific treatment in adult patients with a *de novo* MCD diagnosis. Strikingly, plasmablast percentage tended to be higher in anti-nephrin positive patients, and decreased significantly after 8 weeks of steroid treatment. Of interest, we found a trend toward, but not statistically significant, positive correlation between plasmablasts percentage and anti-nephrin antibodies titers at the time of initial presentation, which highlights the hypothesis of a biological link between plasmablast and anti-nephrin production. Furthermore, transitional B cells and marginal-zone B cells were significantly affected by steroid therapy. Previous extensive B-cell phenotype in patients with INS yielded distinct results depending on the type of cohort (adult vs. children) and the conditions of analysis (previous long history of multitreated INS, relapse vs. remission with or without specific treatment) and genetic background.^3^ Consistent with our results, Oniszczuk *et al.*^15^ found that the percentage of plasmablasts was significantly higher in immunosuppression-naïve adult MCD patients at the time of a first episode of MCD than in nonmatched patients with MCD in remission (with no specific treatment for ≥ 6 months). A similar result was found by Yang *et al.*^16^ among 94 children with primary nephrotic syndrome compared with healthy matched controls. However this finding has not been confirmed in a pediatric cohort at disease onset^17^; the same team showed increased levels of total CD19+, transitional and memory B cells in children with steroid-sensitive nephrotic syndrome at disease onset before immunosuppressive treatment.^18^ Plasmablasts are short-lived activated B cells that may migrate from germinal centers to peripheral blood and later differentiate into antibody-producing plasma cells.^19^ Thus, our findings of increased plasmablast levels in anti-nephrin positive patients at the time of diagnosis as well as their decrease in parallel with disappearance of anti-nephrin antibodies in response to steroid therapy support an antibody-mediated etiology and the use of B-cell–targeting therapies in patients with anti-nephrin antibody positive MCD. In experimental models and in patients with immunological disorders, steroid therapy seems to inhibit differentiation of B cells into plasmablasts and subsequently plasma cells.^20^, ^21^, ^22^ We can hypothesize that short-lived, peripheral, autoreactive plasmablasts are involved in the immunological processes underlying MCD, which are sensitive to the action of steroids. In addition, B-cell depletion after rituximab therapy, which represents a therapeutic approach largely used for treating adult patients with steroid-dependent or frequently relapsing disease,^9^ affects short-lived plasmablasts (present in the periphery) through precursors depletion but not long-lived antibodies secreting cells (bone marrow–residing cells).^22^ This finding may explain the successful use of rituximab as first line therapy in some patients with MCD^23^^,^^24^ and are in agreement with the study of Al Aubodah and colleagues showing elegantly a role for extrafollicular B cells in INS pathogenesis.^25^ Of interest, patients with pemphigus vulgaris share several immunological features with MCD such as an antibody-mediated mechanism directed against intercellular targets, a frequent relapse rate, and a rapid response to immunosuppressive therapy. Cao *et al.*^26^ found that > 75% of patients receiving rituximab and short course of steroid displayed a rapid clinical remission associated with an early depletion of CD20^+^ and CD19^+^ B cells, including plasmablasts; and a marked decrease in circulating autoantibody levels between weeks 4 and 8. This parallel further supports the concept that the kinetics of antibody disappearance in MCD may reflect a corticosteroid- and B-cell–sensitive process, similar to other antibody-mediated diseases such as pemphigus. Interestingly, anti-nephrin negative patients did not show an increase in circulating plasmablasts, with levels remaining unchanged from t_0_ to t_1_. Although the presence of another, as yet unidentified, autoantibody could be hypothesized, the lack of plasmablasts expansion could suggest a different underlying pathophysiological mechanism compared to anti-nephrin positive patients. Notably, 3 out of 5 of these patients responded to corticosteroids, indicating a probable immunological mechanism. Altogether, these findings could suggest that disease activity in this subgroup may be driven by a non–antibody-mediated mechanism, such as T-cell–induced podocyte injury. These observations support the hypothesis that MCD encompasses immunologically heterogeneous forms with differing underlying pathophysiological processes. However, it cannot be fully excluded that possibly unrecorded, distinct temporal dynamics of disease onset may influence plasmablast kinetics in anti-nephrin negative patients. Availability of detailed data on symptom onset and timing of sampling would be valuable to further delineate the temporal relationship between anti-nephrin antibody production, plasmablast expansion, and symptom onset. Moreover, recent therapeutic strategies using anti-CD38–depleting agents (e.g., daratumumab) have shown potential benefits in childhood INS as in adult cases.^27^^,^^28^ Given that CD38 is also expressed on plasmablasts, the therapeutic effect may target both long-lived plasma cells and plasmablasts. Considering that our study analyzed peripheral lymphocyte populations, the characterization of tissue-resident B cells including further characterization of long-lived plasma cells and their role in disease development would be an important next step.

Because the RIFIREINS study is still ongoing, we cannot yet conclude about the potential relationship between the treatment modality, the risk of relapse, the immune reconstitution processes, and the reappearance of antibodies. In additiona, the sample size of the analyzed cohort restricts the power of further statistical analyses.

In conclusion, our prospective study confirms the prevalence of anti-nephrin antibodies in 70% of adult patients naïve MCD at disease onset before immunosuppressive therapy and demonstrates a strong correlation between rapid remission of nephrotic syndrome upon 8 weeks of steroid treatment, disappearance of anti-nephrin antibodies, and a significant reduction of plasmablast levels. Further studies are needed to decipher the precise relationship between B-cell subset reconstitution, the role of tissue-resident B lymphocytes, the reappearance of anti-nephrin antibodies, and the subsequent risk of relapse.

## Disclosure

FE, TBH, and NMT report a pending patent in relation to the measurement of anti-nephrin antibodies. FEH reports honoraria from Novartis. TBH reports consulting fees from Boehringer Ingelheim, Novartis, Alexion, Pfizer, Retrophin-Travere, and Fresenius Medical Care. NMT reports consulting fees from Merida Bioscience and honoraria from CSL Vifor and AstraZeneca. VA received consulting fees from Addmedica, Vifor, Alnylam, and AstraZeneca outside of the submitted work. AK received consulting fees from Roche. All the other authors declared no competing interests.

## Supplementary Material

Supplementary File (PDF) **Figure S1.** Flow chart of patients with available serum samples at the 2 study time points: t_0_, at diagnosis before steroid initiation, and t_1_, after 8 weeks of steroid therapy. **Figure S2.** Gating strategy for the flow cytometry analysis of B-cell subsets. **Figure S3.** Antinephrin autoantibody analysis using immunoprecipitation enzyme-linked immunosorbent assay (IP-ELISA) in patients with minimal change disease.

## Footnotes

Supplementary File (PDF)
**Figure S1.** Flow chart of patients with available serum samples at the 2 study time points: t_0_, at diagnosis before steroid initiation, and t_1_, after 8 weeks of steroid therapy. **Figure S2.** Gating strategy for the flow cytometry analysis of B-cell subsets. Lymphocytes are gated on low-side scatter CD45+ cells. Total B cells are gated on CD19+ lymphocytes. The different B-cell subsets studied were defined and gated on the basis of their expression of IgD and CD27, along with CD38 and CD24. DN, double-negative (IgD- CD27-); MZL, marginal zone-like (IgD+ CD27+); N, naïve (IgD+ CD27-); Pb, plasmablast (IgD- CD27++ CD24- CD38++); SM: switched memory (IgD- CD27+); T, transitional (CD24++CD38++). **Figure S3.** Anti-nephrin autoantibody analysis using immunoprecipitation enzyme-linked immunosorbent assay (IP-ELISA) in patients with minimal change disease. Anti-nephrin levels at diagnosis (t_0_) and after 8 weeks of steroid therapy (t_1_) (A) in patients who were anti-nephrin positive and (B) in patients who were anti-nephrin negative by IP-Western blot at diagnosis. Data are presented as boxplots with medians and interquartile ranges. (C) Correlation between plasmablast percentage and anti-nephrin levels (IP-ELISA, RU/ml) at diagnosis in patients with *de novo* minimal change disease. Spearman correlation: *r* = 0.45, *P* = 0.06.

## References

[1] Saleem M.A. (2019). Molecular stratification of idiopathic nephrotic syndrome. Nat Rev Nephrol.

[2] Vivarelli M., Massella L., Ruggiero B., Emma F. (2017). Minimal change disease. Clin J Am Soc Nephrol.

[3] Colucci M., Oniszczuk J., Vivarelli M., Audard V. (2022). B-cell dysregulation in idiopathic nephrotic syndrome: what we know and what we need to discover. Front Immunol.

[4] Watts A.J.B., Keller K.H., Lerner G. (2022). Discovery of autoantibodies targeting Nephrin in minimal change disease supports a novel autoimmune etiology. J Am Soc Nephrol.

[5] Hengel F.E., Dehde S., Lassé M. (2024). Autoantibodies targeting Nephrin in podocytopathies. N Engl J Med.

[6] Hengel F.E., Dehde S., Kretz O. (2025). Passive transfer of patient-derived anti-nephrin autoantibodies causes a podocytopathy with minimal change lesions. J Clin Invest.

[7] Kidney Disease: Improving Global Outcomes (KDIGO) Glomerular Diseases Work Group (2021). KDIGO 2021 clinical practice guideline for the management of glomerular diseases. Kidney Int.

[8] Hengel F.E., Dehde S., Yilmaz A. (2025). Anti-nephrin autoantibodies in steroid-resistant nephrotic syndrome may inform treatment strategy. Kidney Int.

[9] Gauckler P., Matyjek A., Kapsia S. (2025). Long-term outcomes of rituximab-treated adult patients with podocytopathies. J Am Soc Nephrol.

[10] Al-Aubodah T.A., Piccirillo C.A., Trachtman H., Takano T. (2025). The autoimmune architecture of childhood idiopathic nephrotic syndrome. Kidney Int.

[11] Shu Y., Huang J., Jiang L. (2025). Anti-nephrin antibodies in adult Chinese patients with minimal change disease and primary focal segmental glomerulosclerosis. Kidney Int.

[12] Raglianti V., Angelotti M.L., Cirillo L. (2024). Anti-slit diaphragm antibodies on kidney biopsy identify pediatric patients with steroid-resistant nephrotic syndrome responsive to second-line immunosuppressants. Kidney Int.

[13] Shirai Y., Miura K., Ishizuka K. (2024). A multi-institutional study found a possible role of anti-nephrin antibodies in post-transplant focal segmental glomerulosclerosis recurrence. Kidney Int.

[14] Batal I., Watts A.J.B., Gibier J.B. (2024). Pre-transplant anti-nephrin antibodies are specific predictors of recurrent diffuse podocytopathy in the kidney allograft. Kidney Int.

[15] Oniszczuk J., Beldi-Ferchiou A., Audureau E. (2021). Circulating plasmablasts and high level of BAFF are hallmarks of minimal change nephrotic syndrome in adults. Nephrol Dial Transplant.

[16] Yang X., Tang X., Li T. (2019). Circulating follicular T helper cells are possibly associated with low levels of serum immunoglobulin G due to impaired immunoglobulin class-switch recombination of B cells in children with primary nephrotic syndrome. Mol Immunol.

[17] Zotta F., Vivarelli M., Carsetti R., Cascioli S., Emma F., Colucci M. (2022). Circulating plasmablasts in children with steroid-sensitive nephrotic syndrome. Pediatr Nephrol.

[18] Colucci M., Carsetti R., Cascioli S., Serafinelli J., Emma F., Vivarelli M. (2019). B cell phenotype in pediatric idiopathic nephrotic syndrome. Pediatr Nephrol.

[19] Schrezenmeier E., Jayne D., Dörner T. (2018). Targeting B cells and plasma cells in glomerular diseases: translational perspectives. J Am Soc Nephrol.

[20] Yan S.X., Deng X.M., Wang Q.T., Sun X.J., Wei W. (2015). Prednisone treatment inhibits the differentiation of B lymphocytes into plasma cells in MRL/MpSlac-lpr mice. Acta Pharmacol Sin.

[21] Lanzillotta M., Della-Torre E., Milani R. (2019). Effects of glucocorticoids on B-cell subpopulations in patients with IgG4-related disease. Clin Exp Rheumatol.

[22] Crickx E., Weill J.C., Reynaud C.A., Mahévas M. (2020). Anti-CD20-mediated B-cell depletion in autoimmune diseases: successes, failures and future perspectives. Kidney Int.

[23] Guan N., Zhang M., Zhang M., Chen R., Xie Q., Hao C.M. (2023). Rituximab as initial therapy in adult patients with minimal change disease. Kidney Int Rep.

[24] Xu R., Hu H., Xu H. (2024). Initial rituximab monotherapy for adult indiopathic nephrotic syndrome with minimal change lesion pattern. Nephrol Dial Transplant.

[25] Al-Aubodah T.A., Aoudjit L., Pascale G. (2023). The extrafollicular B cell response is a hallmark of childhood idiopathic nephrotic syndrome. Nat Commun.

[26] Cao S., Yang B., Wang Z. (2025). Efficacy, safety, and B-cell depletion capacity of 3 rituximab dosing regimens in the treatment of moderate-to-severe pemphigus vulgaris and pemphigus foliaceus: a 52-week clinical trial. J Am Acad Dermatol.

[27] Angeletti A., Bin S., Kajana X. (2024). Combined rituximab and daratumumab treatment in difficult-to-treat nephrotic syndrome cases. Kidney Int Rep.

[28] Dossier C., Prim B., Moreau C. (2021). A global antiB cell strategy combining obinutuzumab and daratumumab in severe pediatric nephrotic syndrome. Pediatr Nephrol.

