# Supplementary material for: Effect of Steroids on Anti-nephrin Autoantibodies and B cells in Minimal Change Disease
Source: Kidney Int Rep. 2026 Jan 23;11(4):103794. doi: 10.1016/j.ekir.2026.103794 (PMC12926970; doi:10.1016/j.ekir.2026.103794)
Supplement: Supplementary File (PDF) — Figure S1. Flow chart of patients with available serum samples at the 2 study time points: t0, at diagnosis before steroid initiation, and t1, after 8 weeks of steroid therapy. Figure S2. Gating strategy for the flow cytometry analysis of B-cell subsets. Figure S3. Antinephrin autoantibody analysis using immunoprecipitation enzyme-linked immunosorbent assay (IP-ELISA) in patients with minimal change disease. [file mmc1.pdf]

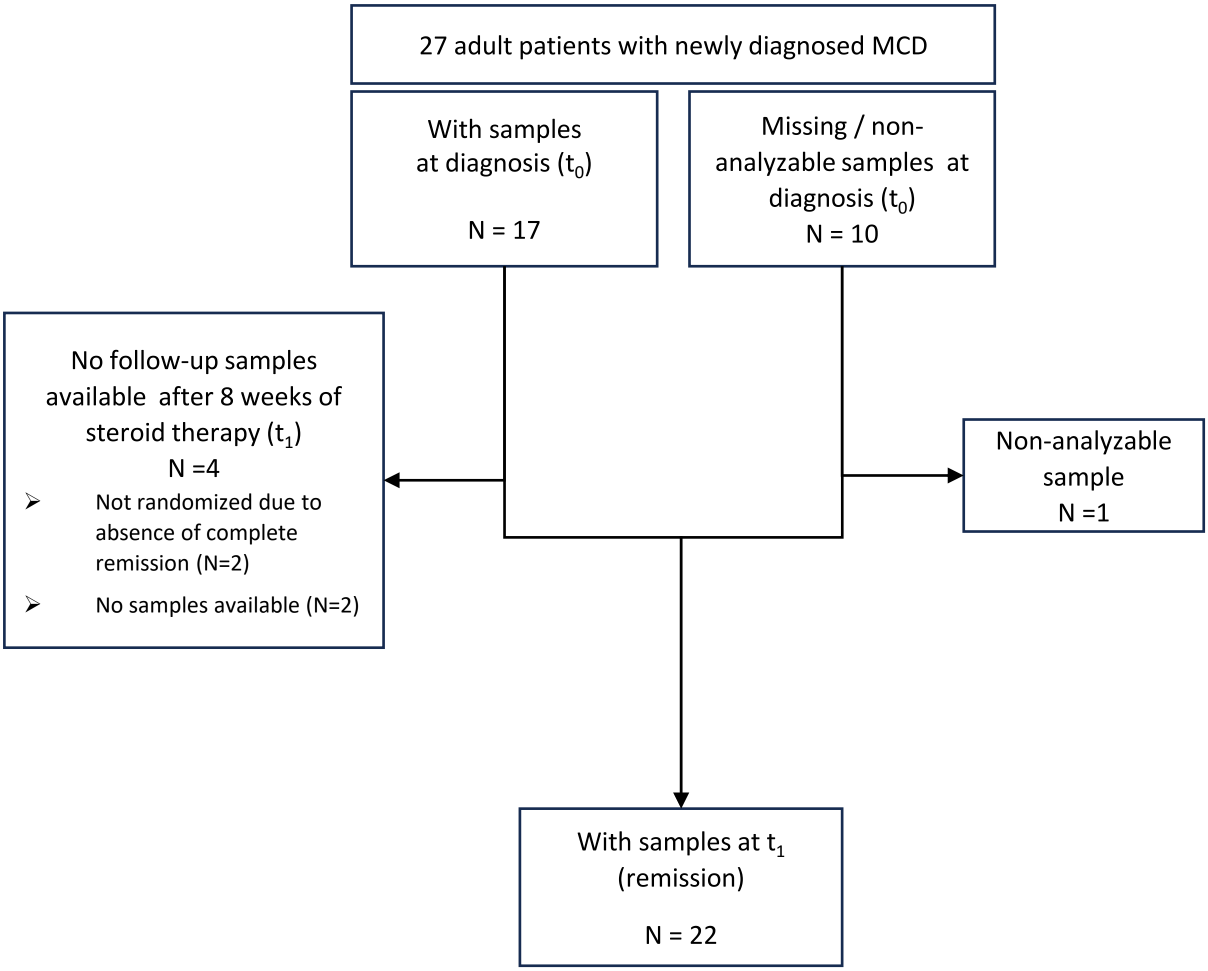

Supplementary Figure S1. Flow chart of patients with available serum samples at the two study time points: t_0, at diagnosis before steroid initiation, and t_1, after 8 weeks of steroid therapy. (MCD: Minimal change disease)

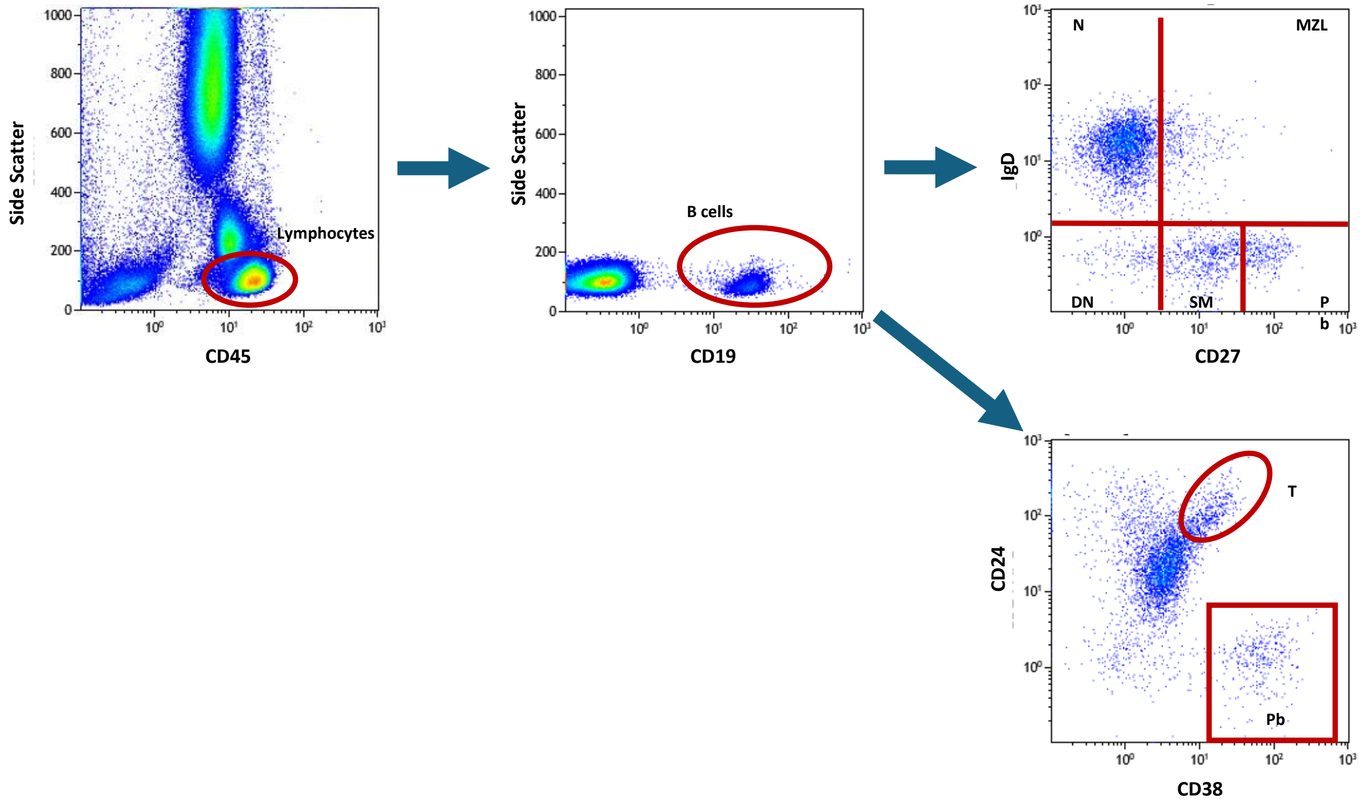

Supplementary Figure S2 Gating strategy for the flow cytometry analysis of B-cell subsets. Lymphocytes are gated on low-side scatter CD45+ cells. Total B cells are gated on CD19+ lymphocytes. The different B-cell subsets studied were defined and gated on the basis of their expression of IgD and CD27, along with CD38 and CD24. N: Naïve (IgD+ CD27-), MZL: Marginal Zone-like (IgD+ CD27+) , SM: Switched Memory (IgD- CD27+), DN: Double-Negative (IgD- CD27-), T: Transitional (CD24++CD38++), Pb: Plasmablast (IgD- CD27++ CD24- CD38++).

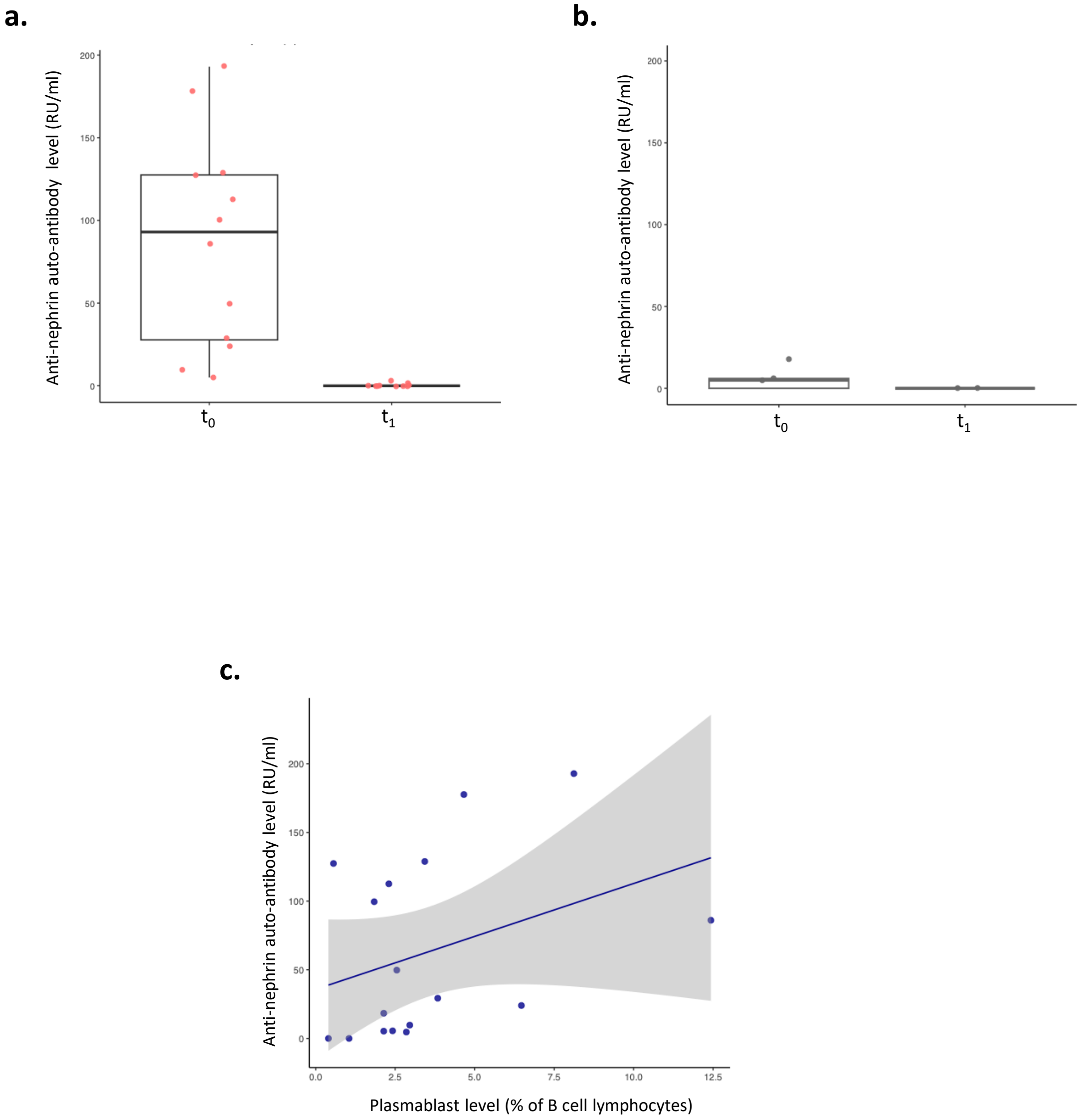

Supplementary Figure S3. Anti-nephrin auto-antibody analysis using Immunoprecipitation enzyme-linked immunosorbent assay (IP-ELISA) in patients with minimal change disease. Anti-nephrin levels at diagnosis (t_0) and after 8 weeks of steroid therapy (t_1) in patients who were anti-nephrin positive (**a**) and in patient who were anti-nephrin negative (**b**) by IP-Western blot at diagnosis. Data are presented as boxplots with medians and interquartile ranges. (**c**) Correlation between plasmablast percentage and anti-nephrin levels (IP-ELISA, RU/mL) at diagnosis in patients with de novo minimal change disease. Spearman correlation: $r = 0.45$, $p = 0.06$.
